# On the Causes, Nature, Pathology and Treatment of a Comparatively New Disease—Malarial Hematuria

**Published:** 1875-12

**Authors:** William N. Bruce

**Affiliations:** Bainbridge, Ga.


### ON THE CAUSES, NATURE, PATHOLOGY AND TREATMENT OF A COMPARATIVELY NEW DISEASE---MALARIAL HEMATURIA.

By WILLIAM N. BRUCE, M.D., of Bainbridge, Ga.

It has devolved on me to present to you an article "On the Causes, Nature, Pathology and Treatment of a Comparatively new Disease—Malarial Hematuria." We have no full details of it in any of our standard text books, and it has only been the subject of discussion in our periodicals for twelve or fifteen years. At this stage of our knowledge of the disease I cannot reasonably be expected to present you with a complete and exhaustive article on the subject. I made no notes at the bedside, and have never made a post mortem examination, and must therefore rely on memory for my facts and attending circumstances. Much, then, that would have been instructive and interesting in the investigation of the subject must be left unexplained for the present.

The striking characteristics of the disease have made a strong impression on the popular mind, while its fatality has stimulated our profession to commendable efforts to gain from every available source everything calculated to throw light on the subject. It is undoubtedly of malarial origin, and confined to localities infected with miasm. It has not been met with in all such places, and we are led to believe that it is rather in such as from which it is never entirely absent. When the winters are very mild and frosts proportionately light, where vegetation is scorched, not killed, by the cold, and where miasm continues to impress its subjects from one season to another without being checked by the intervening winter, are the times in which it prevails. There seems to be little difference now as to who may be the subjects of this disease, and under what conditions they may be attacked.

Dr. Johnson, of Fort Gaines, who has published his views on the subject, uses language which is pertinent, and I will quote and indorse to that extent. He says: "The only persons who are liable to be attacked by malarial hematuria are those who, for weeks, or months, have suffered with frequent relapses of intermittent fever, and neglected to arrest them until several paroxysms have taken place, followed by relapses every few days, until the chills assume a chronic type, manifested by symptoms of general cachexia, indicated by derangements of the chylo-foietie viscera—such as sallow complexion, enlarged, torpid, or otherwise diseased liver; enlarged, indurated, inflamed spleen; capricious, morbid, appetite; lassitude, want of energy, and general cadaverous appearance of the patient." And although post mortem examinations of the viscera, analysis of the blood, of the urine and other secretions with microscopic examinations, are necessary to prove these assertions, leaving nothing to hypothesis or conjecture, the rational signs are sufficiently plain to warrant us in indorsing all that has been asserted of the structural alterations in all the glands, and more particularly the spleen. It is not to be forgotten a moment that no one in robust health ever becomes at once a subject of the symptoms that constitute malarial hematuria. The structural changes must first have taken place; the secretion must have been changed and perverted; excrementitious matter, that should have been eliminated, must have been retained in the circulation; the blood damaged, retaining and diffusing poisonous elements through the whole body. While it is also deficient in those elements necessary to nutrition, the whole system, adynamic and anæmic in character, approaches to something like a living decomposition. If the patient in this state remains exposed to the continued influence of malarial poison, other influences are brought to bear, and another series of phenomena is developed and added to those already existing; nausea and vomiting, and other evidences of hyperæmia, or inflammation, jaundice and hemorrhages make their appearance, with a train of nervous symptoms, which, in the aggregate, go to form the disease, malarial hematuria. If quinine be given in this state of the system which precedes, and is necessary to the development of the disease, it will be followed in many persons immediately by frequent rigors, icterode skin and hematuria—the very symptoms which are considered characteristic of malarial hematuria. I have not seen this statement distinctly made by any one else, but I have verified the fact over and over in the last ten years, and have seen the same effect repeatedly in the same person. We consider the disease as fairly ushered in when jaundice and hematuria, the marked symptoms which give the name and are characteristic, have made their appearance. But preceding these, there has been generally increased indisposition, nausea, anorexia, more or less febrile symptoms, pain in the lumbar and pubic regions. There is a return of irregular, chilly sensations, sometimes followed by very hot skin, quick, though not strong, pulse. Except in a single case, I have never seen anything like distinct periodic remissions. The skin at the same time, from the palest tinge of yellow, gradually deepens in color until it assumes, as the disease advances, a dark saffron or lemon hue, the color keeps pace in intensity with the other symptoms, and is thus of diagnostic value. When the skin begins to assume its own proper hue there is a corresponding improvement in all other respects. This cannot be considered invariable. That the yellow skin is caused by bile reabsorbed into the circulation cannot be reasonably doubted. The effect is the same. Coincident with the jaundice, there will be observed a deeper color in the urine, attended with increased irritability of the bladder, and a temporary increase of urine; dark, coffee-ground looking flocculi are seen floating, then settling in the urine; and in severe attacks I have seen, at my first visit bright red florid blood flowing from the urethra. Some have doubted if this is blood, drawing nice distinctions; and others have suggested that it is a vicarious discharge of bile from the kidneys. Observation and reasoning alike go to prove that it is blood. Damaged blood, it is true, but nevertheless the same fluid that is in the circulation. Like yellow fever, purpura hemorrhagica, scorbutus, etc., this is a hemorrhagic disease, and in that has a strong line of demarkation between it and other fevers. Bilious remittent and intermittent hemorrhage is not confined to the bladder, merely passing off the aborted red globules as some assert. Hæmaturosis occasionally occurs, and hæmoptisis is frequent. It is essentially a hemorrhagic disease, and an inevitable sequence of some of the lesions and consistent with them. In testing with a clean linen rag, I discovered the peculiar blood stain in the first stage, and both blood and bile when the case was convalescing. The third symptom in our enumeration, but first in is debilitating, harrassing, effects on our patient, first in diagnostic value, as directing our attention to the duodenal inflammation existing, first in practical importance, as materially interfering with the exhibition of our remedies, is the incessant nausea, vomiting and thirst. You are continually being called on to mitigate this group of symptoms. Ice, effervescents, acids, alkalies, absorbents are all tried, and none afford more than temporary relief. It is to the presence of this symptom, simultaneous with icterus, which gives us authority to diagnose the existence of a duodenitis without the proofs afforded by a post mortem examination. Flint says: "When anorexia, nausea, vomiting, and tenderness over or in the neighborhood of the epigastrium, coexist with jaundice, we must infer a duodenitis." We find some tenderness but no acute pain over the epigastrium. The pulse, in a majority of my cases, did not present anything remarkable; but I have seen it as low as forty to the minute, with respiration from nine to eleven; and again as high as one hundred and forty to the minute, with respiration thirty-six to thirty-eight. Yet there is nothing contradictory or incompatible in these apparently antagonistic symptoms.

Dr. Johnson gives a somewhat different account of the inception of this disease, and I will quote him again: "The chill or ague is suddenly ushered in with severe shaking rigors, especially in violent attacks. These rigors are marked by an intensity of degree far beyond those which occur in ordinary chills, and in many cases are prolonged much beyound the time generally occupied by the cold stage of an intermittent fever."

Dr. Mitchell, of New Orleans, an extract of whose paper I have seen, describes it as a "disease due to malaria, which is ushered in by a chill of the congestive type, followed by high fever, parched mouth, extreme thirst, suffused eyes, restlessness and anxiety, sighing respiration, quick bounding pulse, during the febrile manifestations, nausea and vomiting are extreme, the patient vomiting a dark grumous bile. After the nausea and vomiting have continued for five or six hours, jaundice and bloody urine show themselves; the suffused eyes now become instantaneously yellow; the red skin changes to a bronzed yellow; with the hematuria we have pain in the back, well defined in the region of the kidneys; the tongue varies in appearance from a pale milky to a straw color, and the skin a dark bronze. After a time a remission occurs, in which there is an abatement of all the symptoms but the nausea and vomiting." I have seen only one case that corresponded generally with his description. Dr. Turpin, of Alabama, after describing two cases of similar character, which he treated, and successfully, with quinine, iron and lime, remarks of them: "I had never before and have never since seen a case of malarial hematuria of a distinct and intermittent type." Even before Broussais directed attention to the universality of gastro enteritis, as the effect of malarial poison, and asserted it to be the sole cause of fever, and explained the universal connection between jaundice and duodenitis, until the present time, nausea and vomiting, and other signs of gastic disorder are generally observed as prominent symptoms of all malarial fevers, remittent and intermittent; and that the more frequent the relapses the more evident does the presence of a duodenitis become. Coexisting, which gradually increases in intensity with each return of the paroxysms, not so apparent to the eyes, or to our rational perceptions, these symptoms are as obvious as are the jaundiced skin and hematuria; and together with icterus establishes the universally accepted fact that inflammation has extended into the ductus choledochus, closing it. Bile is reabsorbed and carried into the circulation, thus inducing another series of symptoms belonging to the disease under consideration, and also the causation of other morbid phenomena of the gravest character, thereby complicating it still farther. Flint says: "Bile in the blood acts as a sedative on the circulation and nervous system. The heart's action is usually diminished in frequency. The pulse frequently falls from fifty to forty, or even much lower in cases in which the jaundice is not associated with acute inflammation or fever. If thus associated the pulse is less effected than if jaundice did not coexist. The respiration is diminished in frequency in proportion to the effect on the circulation. If this condition be prolonged hemorrhages are apt to occur." Again he says: "Bile retained causes debility, nausea," etc. Carpenter says: "That the proximate element of the bile, accumulates in the blood, when from any cause the secretion is suspended, is a fact now well ascertained, and this satisfactorily accounts for the disturbance of other functions, especially those of the nervous system, which then ensues. When the suppression is complete the patient suddenly becomes *jaundiced*, the powers of the system become speedily lowered, (almost as by a narcotic poison), *and death rapidly supervenes.* When the secretion is diminished (not suspended) the same symptoms present themselves in a less aggravated form." But at the stage at which we meet it the kidneys are already involved enough to have a hematuria, and may become so affected as to lead to uræmia. Dr. Mitchell found traces of extreme disease of the kidneys in his post mortem examinations. The kidneys in their attempt to throw off vicariously the various irritating excrements retained in the blood, with a hemorrhagic diathesis existing, have become inflamed and enlarged, and have hematuria, and if this diseased state is sufficient to interfere materially with the due performance of its functions, and if the secretion is suspended or much diminished, uræmia is necessarily the consequence, the last, most fatal of the symptoms, sometimes, not always, met with in malarial hematuria. The retention of a small amount of urea is not incompatible with health, since it exists normally in the blood. The presence of a large amount is not compatible with health, but not necessarily fatal; but when most of it is retained, as in the case of complete or partial suppression, death speedily supervenes. We have no other means, except analysis of the blood, of ascertaining if there is a dangereous amount of urea in the blood; but this should warn us to take means to meet the emergency, and when an intensely hot skin and quick pulse is found coexisting, with jaundice and partial suppression of urine, with no local inflammation to account for it, you may safely diagnose and treat it promptly as such.

*Treatment.*—Before we enter on the subject of treatment, we will briefly recapitulate the group of symptoms and condition, which we are to prescribe for under the name of malarial hematuria.

1. Chronic enlargements of the spleen, and perhaps of the liver. 2. Sub-acute inflammation of the duodenum, which extending causes, 3. Jaundice or reabsorption of bile into the circulation, in its turn inducing the hemorrhagic diathesis, damages the blood, reduces the heart's action and the number of respirations in a marked degree, and induces a train of remarkable nervous symptoms. 4. Inflammation of the kidneys, accompanied with hematuria, may cause a suppression, and lead to uræmia, an excess of urea being a prompt, energetic poison. 6. Anemia, a want of the proper proportions in the blood, a deficiency of red globules and an excess of white, thus arresting nutrition and impoverishing the blood.

These conditions it is our object to restore to a healthy state, by appropriate remedies, so selected that we understand the rationale of their action, and the distinct effect that each is to produce. A great many remedies, having different and even antagonistic effects, have been recommended. A great many combinations have been used in the treatment, leaving it difficult to select those from which benefit has been derived. We have no means yet of comparing their mixed and conflicting effects; but we keep before our minds the abnormal conditions we are to treat or restore to health. Unquestionably the nausea and vomiting and jaundice point us first to the relief of the gastro-duodenitis, the cause of the symptoms and the distressing nervous phenomena which accompany them. The patient will require relief at your hands, and until you soothe the irritable stomach for a time you are almost arrested in your treatment. A full laxative effect on the bowels is required at once, and continued moderately throughout the treatment. While anti-emetics of every kind are being tried, synapisms, ice, effervescents, acids, absorbents, may all be resorted to, and lastly the salts of morphia. Dry cupping over the epigastrium gives notable relief. Of the many remedies to relieve nausea ice, sub. nit. bismuth in large doses, and champagne, have most frequently proved efficient; but it is on morphia, given at regular intervals, that we are to rely with confidence, as it will never fail. I prefer some one of the effervescing salts as a purgative, aided by cold enemata. Calomel seems to be more generally prefered by all who have published their views. If we could see its laxative, without danger of its mercurial effects, there would be considerable advantage in its use, being less offensive to the stomach and not easily rejected; but there is considerable danger of its specific effect, which is directly contra-indicated in the state of the system under consideration. I severely salivated one of the only two patients to whom a mercurial was given by a single blue pill, and I nferred that there is more than ordinary susceptibility to mercurial impression in them; but it is antiphlogestic in its effects, and every condition and symptom of malarial hematuria, adynamic anæmia and asthenia require the very reverse. Flint says mercurialization reduces the number of red globules in the blood, and their deficiency being one of the main features in these lesions, and their restoration is, of course, one of the main objects in the treatment. Another reason for the rejection of calomel is the fact that I administer the acids, especially nitric acid, through every stage of the disease, and there is a general belief even among the profession that the acids, exhibited with mercury, predispose to ptyalism. Ice in small quantities is to be allowed to the patient *ad libitum*; if it is not to be had, cold water instead; and here I protest against the unconditional condemnation of cold water made by so high an authority as Dr. Johnson. He says: "Cold water taken even in the smallest quantities proves a violent irritant to the stomach, which will be rejected soon after it is swallowed, preceded by strong and straining efforts, retching, and therefore we must enjoin the most absolute abstinence from the use of this and other fluid substances, until we get this troublesome and distressing symptom under control." I object *in toto* to the above. It is true that sometimes we must use a prudent discretion in abstaining from everything for a time in very obstinate vomiting; but in rejecting ice and very cold water, we are surrendering our most efficient remedies in a most troublesome emergency, and adding immensely to the sufferings of our patient. Not only is it necessary to quench the thirst of our patient, but we are to *dilute* the fluids in the circulation and in the secretions. Large quantities of water, if they can be retained, should be taken in order to increase and dilute the urinary secretion, loaded with irritating excrementitious matter. The arrest or partial suspension of this is a most dangerous feature. Some time during the past year I read an abstract of an address, delivered before an association in North Carolina, in which its author condemns the use of acids in malarial hematuria. He asserts that the system is already overcharged with acids, and that we are aggravating the disease, etc. I have not analyzed the fluids of the body, but if it be true that soda enters largely into the composition of the bile, and is diffused over the body in the circulation, and, Flint says, separated from its usual acid combination, I fail to understand how the fluids can be acid. If the disease is prolonged to a later stage, in which uræmia exists, then carb. ammonia is present in excess, and we are not convinced of the presence of acids. We do know that acids are more grateful to the patient than any thing alkaline; that the patient improves or recovers under their use. We can understand why the veteran, Chapman, prescribed lemonade and hard cider for jaundice. I give nitric acid in every stage of the disease, in twenty to thirty drops a day. I have used mur. tincture of iron in the same way, and I have seen nothing to abate my confidence in their use. Nitric acid has had a very high reputation a long time as a remedy in the chronic diseases, enlargments, etc., of the liver and spleen in hot climates. From the very happy influence that it exerts in some diseases of the intestinal canal, combined with opium, dysentery, etc., we are encouraged to expect in similar combinations good results in duodenitis, and are not disappointed in practice. It increases the proportion of red globules and diminishes the white, thus renovating the blood. It is tonic and alterative. It decomposes urea in the circulation, and may thus protect from uræmic poisoning. We have already alluded to the necessity of resorting to opiates to allay the irritability of the stomach. It is our most valuable remedy, not only for this purpose, but is beneficent in controlling the whole train of nervous symptoms, with the circulation, reducing the pulse in twenty-four hours from one hundred and forty to one hundred and ten beats We are told by our authorities that "it soothes the inflamed surface in duodenitis," and from the remarkable influence opium exerts over inflammatory affections of the bowels, we have a right to suppose duodenitis is in like manner benefited by it. It is indispensable in the treatment of inflammation of the kidneys and bladder and when uræmia sets in it renders the system tolerant of that poison, until time is gained to make an effort to eliminate it from the circulation. My word (to use the language of another) without the use of opium and its preparations, all the effects of the physician to control them would prove abortive and unavailing. I rely on it and prescribe it with the confidence that we are wont to place in quinine in intermittents. I have sometimes used hyd. chloral, in its stead, with satisfactory results. A medical friend recommends highly the preparations of gelseminum, and from the combination of anodyne properties, with others over the urinary organs, I should expect good results. The bromide potassium, with its sedative effect on the nervous system, also has a peculiar effect in reducing enlargements of the spleen.

Iodide of potassium is also very much extolled by some. I have always prescribed some preparation of iron during convalescence the anæmic condition of the patient making it indispensable. There are many remedies that under certain circumstances may be indicated. If the gastric symptoms were very obstinate, a blister to the epigastrium might be required, but I should resort to it with reluctance, lest it might increase the irritation of the urinary organs, and remove it before complete restoration takes place. I have never prescribed directly for the hematuria, considering it only a symptom of other lesions, and I have not met with a case in which it seemed to add to the danger. Where you meet a case characterized by an intensely hot skin, quick pulse and respiration, overriding the slow pulse and respiration usually accompanying jaundice, and not satisfactorily to be attributed to a gastric inflammation, especially if there is some dullness of the faculties; if in this state you find a deficiency of urinary secretion, you have uræmia, an occasional, not universal, sequence of these conditions—the most unmanageable and fatal of them all. The most of the symptoms are so common in every form of fever, that you may not attribute them to these proper causes, nor notice the eminence of the danger. It will require some confidence in your correctness of diagnosis, some decision of character, some thing above the fear of blame, to prescribe fearlessly and push as far as may be safe the salts of morphia, even though your patient may be on the verge of coma, and his friends may attribute it to the remedy. But all the authorities and my experience show that nothing better can be done than obtain tolerance to the uræmic poison by opiates, while we are endeavoring to create a vicarious discharge by purgatives, and to restore the diminished urinary secretion. If we fail *death speedily supervenes.* In ordinary cases of malarial hematuria, when the hematuria ceases, and icturus passes away, a *quasi* convalescence takes place. The patient is by no means yet well—he has only partially recovered. There are still enlargements, depraved secretions and damaged blood to be restored. He will be liable to relapse, and never recover health during the existing season, but he should continue a subject of medical supervision until his health is fully re-established.

*Quinine in Malarial Hematuria.*—I might bring this article to a close now with propriety, since it is outside of my subject to comment on remedies that I do not use in the treatment of malarial hematuria, but which are recommended and relied on by a majority of the profession. Their preponderance in standing and in numerical strength indeed places me on the defensive, and although I do not willingly stand almost alone in my opinion, my experience in the disease, my opinion of its true pathology, and of the therapeutical uses of quinine, have brought me to the conviction: first, that quinine is seldom indicated and very rarely if ever beneficial in malarial hematuria; second, that the treatment by quinine has not proved more successful than without it, and therefore it is unnecessary; third, that it is, in some cases, decidedly pernicious; fourth, that in the condition of the system antecedent to malarial hematuria and predisposing to it, quinine produces and develops the symptoms (rigors, jaundice, hematuria) called malarial hematuria. No one who has practiced medicine in this climate can be blind to the fact that it cannot be relied on in the cure of common chronic chills and fever. It generally fails, and if it arrests the paroxysm a single time, in a week or fourteen days there is a relapse, and then it is still less to be relied on; and, if we do resort to it to arrest the ensuing paroxysm, we are compelled to depend on other remedies—iron, arsenic, strychnine—to perfect the cure. In the relapsing, four of our text books (which is the same disease before the jaundice and hematuria have taken place) it is distinctly stated that quinine is unreliable and useless. Every step in the progress of chronic malarial fever, the enlarged spleen and liver, altered secretions, damaged and impoverished blood, anemia, duodenic inflammation, hemorrhagic diathesis to uræmia, removes it further and further from the range and health restoring influence of quinine, which is only useful in acute stages of the disease.

Let us analyze the therapeutical uses of quinine. An abstract, from lectures of Mr. Lee, of London, on the therapeutic effects of quinine, says: "Quinine has a threefold action. It diminishes the frequency and force of the heart's action; it lowers the tension of the arterial system; it lowers the temperature." In malarial fevers, he shows that the drug cannot be relied on as a specific. It does not prevent malarial poison when taken as a prophylactic. It does not prevent recurrence after a variable period, and is useless in some of its most fatal forms. Another article, "On the Uses and Abuses of Quinia, and its salts," being a prize essay by Dr. Bartholomew, of Conn., thus sums up its specific properties: "Quinine is prophylactic, but its power declines with use; cures acute malarial poisoning without influence upon the structural alterations of chronic malarial poisoning, and only effective *temporarily* against the febrile movements; exerts an inhibitive influence on the heart and arterioles, impairs primary assimilation and damages the blood; abuses *in chronic malarial poisoning*, in fevers, in acute rheumatism; as a *tonic* in acute and chronic diseases." Quinine is not antidotal in its effects; it does not neutralize miasmatic poison, but exerts its influence directly on the animal organism, controlling some of the effects of malarial poison, chiefly the undue action of the heart and arteries and the increased temperature of the body. Which one of the group of symptoms constituting malarial hematuria is it designed to meet? What abnormal condition will it correct or restore? The enlarged indurated liver and spleen? But our authorities say that it is "*without influence upon the structural alterations of chronic malarial poisoning.*" It is abused in chronic malarial poisoning or as a *tonic* in acute and chronic diseases. And this is precisely the view I have taken of its uses, and endeavored to express. That it will produce, in certain conditions, both jaundice and hematuria, and aggravate the accompanying symptoms, I am able to prove by numerous sufferers and their friends in this vicinity. My first patient with malarial hematuria asserted that he had a chill and an aggravation of all his symptoms immediately after each dose of quinine, which he had taken in four doses every three hours; but I continued it until I became convinced that his statement was true, and then substituted dover powder and morphine, with satisfactory results. My next patient recovered with no quinine after my first visit, but was sorely salivated by a single blue pill. My third patient, in consultation, died the same night that I saw her. Her medical attendant told me she had taken quinine heroically, and he candidly thought it did harm. I have often been called to patients since under the influence of quinine, and found them more unmanageable than those who had taken none; and, except in a single case, differing in many respects from others, I have not prescribed it since, but have watched it with lively interest in the hands of others. When your attention is once directed to its effects, you may often perceive effects less distinct, and less injurious than then, but still prejudicial; and with a remedy so potent for good, if it has no positively beneficial effect, it is as actively injurious.

